# Placental Fatty Acid Ethyl Esters Are Elevated with Maternal Alcohol Use in Pregnancies Complicated by Prematurity

**DOI:** 10.1371/journal.pone.0126552

**Published:** 2015-05-15

**Authors:** Theresa W. Gauthier, Sowmya S. Mohan, Teresa S. Gross, Frank L. Harris, David M. Guidot, Lou Ann S. Brown

**Affiliations:** 1 Department of Pediatrics, Division of Neonatal-Perinatal Medicine, Emory University School of Medicine, Atlanta, Georgia, United States of America; 2 Department of Medicine, Division of Pulmonary, Allergy, and Critical Care Medicine, Emory University School of Medicine, Atlanta, Georgia, United States of America; Xavier Bichat Medical School, INSERM-CNRS - Université Paris Diderot, FRANCE

## Abstract

The accumulation of fatty acid ethyl esters (FAEEs) in meconium of term newborns has been described as one potential biomarker of maternal alcohol use during pregnancy. FAEEs accumulate in multiple alcohol-exposed fetal tissues and in the placenta. Limited research has focused on the identification of the premature newborn exposed to alcohol *in utero*. We *hypothesized* that maternal alcohol use occurs in a significant proportion of premature deliveries and that this exposure can be detected as elevated placental FAEEs. The goals of this study were to 1) determine the prevalence of maternal alcohol use in the premature newborn and 2) investigate whether placental FAEEs could identify those newborns with fetal alcohol exposure. This prospective observational study evaluated 80 placentas from 80 women after premature delivery. Subjects were interviewed for alcohol intake and placental FAEEs were quantified via GC/MS. Receiver Operator Characteristic (ROC) Curves were generated to evaluate the ability of placental FAEEs to predict maternal drinking during pregnancy. Adjusted ROC curves were generated to adjust for gestational age, maternal smoking, and illicit drug use. 30% of the subjects admitted to drinking alcohol during pregnancy and approximately 14% answered questions indicative of problem drinking (designated AUDIT+). The specific FAEEs ethyl stearate and linoleate, as well as combinations of oleate + linoleate + linolenate (OLL) and of OLL + stearate, were significantly (p<0.05) elevated in placentas from AUDIT+ pregnancies. Adjusted ROC Curves generated areas under the curve ranging from 88–93% with negative predictive values of 97% for AUDIT+ pregnancies. We conclude that nearly one third of premature pregnancies were alcohol-exposed, and that elevated placental FAEEs hold great promise to accurately determine maternal alcohol use, particularly heavy use, in pregnancies complicated by premature delivery.

## Introduction

One of the most reliable direct biological markers of prenatal exposure to alcohol in the term newborn is elevated fatty acid ethyl esters (FAEEs), formed via esterification of alcohol with endogenous free fatty acids. Alcohol is metabolized by both oxidative and non-oxidative pathways [1] and FAEEs are the product of the non-oxidative pathway where alcohol conjugates to free fatty acids [2]. FAEEs have been identified as markers of both acute and chronic alcohol exposure in adults [3,4]. For the term newborn, FAEEs accumulate in meconium with maternal alcohol use during pregnancy [5,6,7,8,9,10] and can predict adverse neurological outcome in the exposed newborn [11,12]. Animal models of fetal ethanol exposure have demonstrated accumulation of FAEEs in multiple fetal tissues including the placenta which contains FAEE synthase activity [7]. FAEE accumulation correlated with pathology in multiple fetal organs [13].

However, limited research has focused on the identification of the premature newborn exposed to alcohol *in utero*. The need for such research was highlighted by a recent study which demonstrated a dramatic 35 fold increased risk of extreme premature delivery in mothers who drank alcohol during pregnancy [14]. Identification of the alcohol-exposed newborn often relies on maternal disclosure of consumption using structured maternal interviews and questionnaires. However, this approach often underrepresents true intake during pregnancy [15,16]. The use of FAEEs to identify the alcohol-exposed premature newborn has not been evaluated.

We therefore *hypothesized* that maternal alcohol use occurs in a significant proportion of premature deliveries and that placental FAEEs would be elevated in pregnancies where maternal alcohol use was reported. The goals of the current study were to evaluate maternal alcohol use in premature newborns delivered at ≤1500 grams birth weight, to determine whether FAEEs were elevated in placental tissue, and to determine if placental FAEEs could be indicative of fetal alcohol exposure.

Our results demonstrate that, per maternal report, approximately one in three premature pregnancies were alcohol-exposed while problematic drinking was reported in one in seven pregnancies. Individual placental FAEEs and combinations of FAEEs were significantly elevated with maternal alcohol use and hold promise to identify the alcohol exposed premature newborn.

## Materials and Methods

### Human participants

This study was approved by the Emory IRB (Emory IRB 00000976, Gauthier, PI) and written informed consent was obtained from all subjects at the time of enrollment. Subjects were enrolled from Emory University Hospital Midtown and Grady Memorial Hospital in Atlanta, GA from 11/2009-12/2012. Mothers of all neonates weighing less than or equal to 1,500 grams who were admitted to the Newborn Intensive Care Units of Grady or Emory Midtown were eligible for enrollment into the study. Exclusion criteria included maternal refusal to participate, multiple congenital anomalies on physical exam, and clinically suspected or confirmed chromosomal abnormality. Mothers whose babies were deemed non-viable by the attending neonatologist were not approached for enrollment. Mothers with maternal HIV history were excluded because of the potential risk to laboratory personnel in the sample handling and analysis.

### Placental Collections

After informed consent, placentas were harvested after delivery using the Human Tissue Procurement Service (Winship Cancer Institute, Emory University). A tissue sample was uniformly collected as a full thickness section from the edge of the placenta, approximately 5 cm from the point of umbilical cord insertion. The sample was frozen at -80°C until batched analysis via GC/MS (Emory + Children’s Pediatric Research Center Biomarkers Core) in the Brown laboratory.

### Maternal Questionnaire

After informed consent, the subjects were interviewed using a structured, extensive questionnaire administered by trained research staff. The standardized questionnaire was modeled after those used by the Centers for Disease Control and Prevention in studies focusing on maternal alcohol use and outcomes of pregnancy [17,18] and in studies of term newborns we have previously reported [19]. The questionnaire incorporated questions modeled after the Alcohol Use Disorders Identification Test (AUDIT) to identify problem drinking [20]. During the interview, the subjects were asked about lifestyle and behaviors including alcohol consumption (beer, wine, or liquor). A calendar was used to assist in maternal timing of alcohol consumption three months before and during pregnancy. Binge drinking was defined as drinking at least five drinks in a single sitting. Subject education, marital status, income, tobacco smoking, and illicit drug usage were also noted. Subjects were identified with a study number and strict confidentiality was maintained. The subject’s medical record was reviewed for maternal demographics and delivery room information. Data were abstracted by study staff into a secure de-identified electronic data base (Emory Alcohol Lung Biology Center, Guidot PI).

### Determination of Placental FAEEs via GC/MS

After collection, the placental samples were labeled with a de-identified study number and stored at -80°C until batch analysis by GC/MS using methods we have previously described [21]. Briefly, thawed placental samples (0.5 gram) were homogenized and spiked with a non-biological surrogate standard (SS) of pentadecanoic acid ethyl ester (MP Biomedicals, LLC, Santa Ana, CA). The FAEEs of interest (ethyl palmitate (16 carbons; no double bonds); ethyl stearate (18 carbons; no double bonds); ethyl oleate (18 carbons; cis-Δ 9); ethyl linoleate (18 carbons; cis,cis-Δ9,Δ12); ethyl linolenate (18 carbons; cis,cis,cis-Δ9,Δ12,Δ15); and ethyl arachidonate (20 carbons; cis,cis,cis,cis-Δ5Δ8,Δ11,Δ14) were extracted using methanol/chloroform as we have previously published [22,23,24]. Samples were filtered across extraction columns (UCT, Bristol, PA), dried under nitrogen gas, and then reconstituted in methanol. A single column GC/MS using a Hewlett-Packard 5890 Series II GC and a Hewlett-Packard 5972A Mass Selective Detector with analysis via Chemstation Productivity Software G1701BA (Version B.01/.01) was used. The internal standard (heptadecanoic acid ethyl ester, 100 μg/ml, Nu-Chek-Prep, Inc., Elysian, MN) was added to all samples and standards before analysis by GC/MS. Individual FAEEs were identified via their unique retention times and confirmed using a calibration standard composed of all FAEEs of interest (1000 μg/ml, Cayman Chemicals, Ann Arbor, Michigan). The concentrations (μg) of the individual FAEEs of interest were normalized to the dry weight of a corresponding thawed placental sample (grams) obtained after drying (48 h at 50°C). The calibration curves of the FAEEs standards demonstrated a linear fit with a mean coefficient of determination (*r* ^2^) ranging from 0.954–0.979, the lower limit of detection (LOD) ranging from 5.99–8.73 μg/ml and the limit of quantification (LOQ) ranged from 8.16–37.1 μg/ml as we have previously described [21].

### Statistical Analyses

Continuous variables were compared using Student’s t-test and categorical variables were compared using the Chi-square or Fisher exact test when appropriate. Since values of placental FAEEs were not normally distributed, non-parametric analysis was performed using the Mann Whitney-U test and ANOVA with Dunnett post hoc analyses was used to compare multiple groups. Logistic regression models to categorize a neonate as either alcohol exposed or unexposed were created based on FAEE concentration, gestational age, maternal smoking, and maternal drug use. The log transformation of the FAEE concentration was used to account for non-normality. Receiver operating characteristic (ROC) curves were generated to characterize the ability of the model to correctly classify alcohol exposure. A p ≤ 0.05 was considered statistically significant. All analyses were performed using SPSS Statistics Version 21 (IBM, Armonk, NY) and SAS Version 9.4 (Cary, NC).

## Results and Discussion

### Subject Demographics

Eighty (80) subjects were enrolled and 80 placental samples were evaluated. Six sets of twins were delivered, however only the twin A placenta was used for the analysis. The majority of subjects were African American (84%) with 11% being Caucasian and the remaining 5% of subjects noting other race including mixed African American/Caucasian. The majority of the women received prenatal care (91%) while only ~18% of the population reported they were trying to get pregnant with the current pregnancy. Most of the subjects underwent Cesarean Section (70%) while the remainder delivered vaginally. By study design, the median gestational age of the premature newborn was 28 weeks (range: 24–35 weeks) with a median birth weight of the newborn was 1060 grams (range 510–1500 grams). 18% of the premature babies were small for gestational age (SGA).

### Maternal Drinking During Pregnancy

Results of the maternal questionnaire revealed that 64% of the subjects reported drinking alcohol three months before pregnancy while 14% reported binge drinking three months before pregnancy. During pregnancy, 30% (24/80) of the subjects reported consuming alcohol at some time during the pregnancy (Designated Drinker) and the remaining 70% (56/80) denied alcohol consumption during pregnancy (designated Non-Drinker). 14% of subjects (11/80) answered any AUDIT question in the affirmative (designated AUDIT +). 5% (4/80) of all subjects reported binge drinking during the pregnancy. Smoking cigarettes during pregnancy was reported in 16% (13/80) of the subjects, while illicit drug use (including marijuana, heroin, methadone, and speed) occurred in ~14% (11/80) of the subjects (Table 1).

**Table 1:** Maternal Characteristics.

Subject Groups (N)		Overall (80)	Non-Drinker (56)	Drinker (24)	Audit+ (11)
**Maternal Age**, median (25^th^-75^th^)		28.1 (22.4–33.7)	25.3 (22.0–32.6)	30.9 (25.2–35.7)	30.6 (27.1–36.7)
**Race**, N (%)	White	9 (11)	7 (13)	2 (8)	2 (18)
	Black	67 (84)	46 (82)	21 (88)	9 (82)
	Other	4 (5)	3 (5)	1 (4)	0 (0)
**Gravidity**, median (25^th^-75^th^)	Total	3 (1.75–4)	3 (2–4)	3 (1–5.75)	3 (1–4)
	Livebirth	2 (1–3)	2 (1–3)	2 (1–3)	2 (1–3)
	Losses	1 (0–2)	0.5 (0–1.75)	1 (0–2)	1 (0–2)
**Prenatal Care**, Yes, N (%)		73 (91)	50 (89)	23 (96)	10 (91)
**Maternal Weight Gain**, Mean (SD) (n = 75)		18 (13)	17 (12)	21 (14)	19 (13)
**Education**, N (%)	< High school	13 (16)	10 (18)	3 (13)	1 (9)
	High school	26 (33)	16 (29)	10 (42)	3 (27)
	College/tech school	27(34)	20 (36)	7 (29)	5 (46)
	Junior college graduate	1 (1)	1 (2)	0 (0)	0 (0)
	College graduate	9 (11)	7 (13)	2 (8)	2 (18)
	Any graduate studies	4 (5)	2 (4)	2 (8)	0 (0)
**Yearly Income**, N (%) (n = 71)	< $25,000	40 (56)	28 (56)	12 (57)	6 (55)
	$25,001-$55,000	21 (30)	14 (28)	7 (33)	5 (46)
	$55,001-$70,000	2 (3)	2 (4)	0 (0)	0 (0)
	> $70,000	8 (11)	6 (12)	2 (10)	0 (0)
**Marital Status**, N (%)	Married	16 (20)	13 (23)	3 (13)	1 (9)
	Single	60 (75)	39 (70)	21 (88)	9 (82)
	Separated/Divorced	4 (5)	4 (7)	0 (0.0)	1 (9)
**Drinking**, N (%)	Before pregnancy	51 (64)	27 (48)	24 (100)†	10 (91)*
	Binge before pregnancy	11 (14)	4 (7)	7 (29)*	5 (46)†
	Drink during pregnancy	24 (30)	0 (0)	24 (100)	7 (64)
	Binge during pregnancy	4 (5)	0 (0)	4 (17)	4 (36)
**Tobacco Smoking**, N (%)		13 (16)	6 (11)	7 (29)*	6 (55)†
**Illicit Drug Use**, N (%)		11 (14)	7 (13)	4 (17)	3 (27)
**Diabetes**, N (%)		8 (10)	5 (9)	3 (13)	1 (9)
**Gestational Diabetes**, N (%)		1 (1)	1 (2)	0 (0)	0 (0)
**Essential Hypertension**, N (%)		27(34)	19 (34)	8 (33)	3 (27)
**Pregnancy Induced Hypertension**, N (%)		15 (19)	10 (18)	5 (21)	1 (9)

*p≤ 0.05 versus Non-drinker,

^†^p≤ 0.005 versus Non-drinker.

The subjects were assigned to three groups based on the answers provided during their extensive interview: 1) Non-Drinkers, 2) Drinkers, and 3) AUDIT + (as defined above). Maternal characteristics were assessed for the entire study population and then Drinkers compared to Non-Drinkers and AUDIT+ compared to Non-Drinkers. There were no significant differences in maternal age, race distribution, gravidity, prenatal care, maternal weight gain, education, income, or marital status between the groups. Maternal smoking was significantly higher in Drinkers (Odds Ratios (OR) 3.4 [CI- 1.01–11.6], p = 0.04), and in AUDIT+ (OR 8.1 [CI-1.9–33.6] p = 0.002) but there were no differences in reported illicit drug use between the groups. There were no differences in diabetes, gestational diabetes, essential hypertension or pregnancy induced hypertension between the groups. There were also no significant differences in route of delivery, prolonged rupture of membranes, gestational age, or percentage of SGA babies between the groups (p = NS, data not shown).

### Placental FAEEs were elevated with maternal alcohol use during pregnancy

Ethyl palmitate, stearate, oleate, linoleate and arachidonate were readily detectable in the placental samples by GC/MS (Table 2). The values [median (25%, 75% interquartile range)] of each ethyl ester and the sum of all placental FAEEs are presented in Table 2 for the entire subject population and for the Non-Drinkers, Drinkers, and AUDIT+ groups. As noted, individual FAEEs such as Ethyl Palmitate and Ethyl Stearate were significantly elevated in the placentas of Drinkers compared to Non-Drinkers, while Ethyl Stearate and Ethyl Linoleate were significantly elevated in the placentas of AUDIT+ compared to Non-Drinkers.

**Table 2:** Fatty Acid Ethyl Ester Profile in Placenta.

	Overall	Non-Drinker	Drinker	Audit+
**Ethyl Palmitate**	216.17 (122.81–308.13)	203.03 (87.13–272.88)	263.65 (202.73–394.13)*	293.8 (211.55–368.47)
**Ethyl Stearate**	64.4 (3.27–104.05)	40.32 (2.14–94.13)	89.27 (49.72–158.67)*	112.29 (76.59–179.09)*
**Ethyl Oleate**	117.07 (40.65–171.25)	108.7 (23.87–156.15)	142.07 (68.97–212.08)	143.87 (115.48–243.12)
**Ethyl Linoleate**	92.16 (5.21–198.02)	73.97 (3.82–188.34)	132.55 (31.05–213.79)	207.52 (128.29–297.04)*
**Ethyl Arachidonate**	425.93 (135.68–1285.07)	393.81 (124.03–1114.10)	528.83 (237.91–1366.89)	624.56 (232.16–1219.07)
**Total FAEEs**	1052.75 (469.86–1846.97)	998.82 (322.11–1676.96)	1396.22 (798.77–2476.76)	1347.75 (870.34–2495.38)

Values are μg/gm dry weight expressed as Median (25^th^ -75^th^). Non parametric comparisons by Mann-Whitney U Test.

*p≤ 0.05 versus Non-drinker.

Placental FAEE levels did not significantly differ by the sex of the offspring (p = NS for each FAEE, data not shown). Interestingly, although there was no difference in race distribution among the drinking groups (Table 1), there were significant differences in ethyl palmitate when comparing White, Black and Other groups (p<0.05, ANOVA). Levels of placental ethyl palmitate in Blacks (Median [25^th^-75^th^]: 224.92 [145.2–359.4] μg/mg dry weight) were no different to Other (261.2 [81.6–281.8] μg/mg dry weight) (p = NS) but were significantly higher than Whites (94.9 [5.6–218.8] μg/mg dry weight) (p<0.05). Placental FAEE levels did not significantly correlate with neonatal birth weight (as assessed by Z-score for gestational age and by percentile for gestational age) (p = NS for each FAEE, data not shown). There was a weak, albeit positive, correlation between ethyl arachidonate and gestational age (Kendall’s tau_b coefficient 0.199; p = 0.016) and total FAEEs and gestational age (Kendall’s tau_b coefficient 0.161; p = 0.05). There were no significant differences in placental FAEEs when comparing cesarean section versus vaginal delivery (p = NS for each FAEE, data not shown). Furthermore, there were no correlations between FAEEs and length of rupture of membranes (p = NS for each FAEE, data not shown), nor were there differences in placental FAEEs in pregnancies with prolonged rupture of membranes >8 hours versus those without prolonged rupture (p = NS for each FAEE, data not shown). Placental chorioamnionitis was reported in only 5% of the samples and there were no differences in FAEEs (p = NS for each FAEE, data not shown).

**Fig 1:**
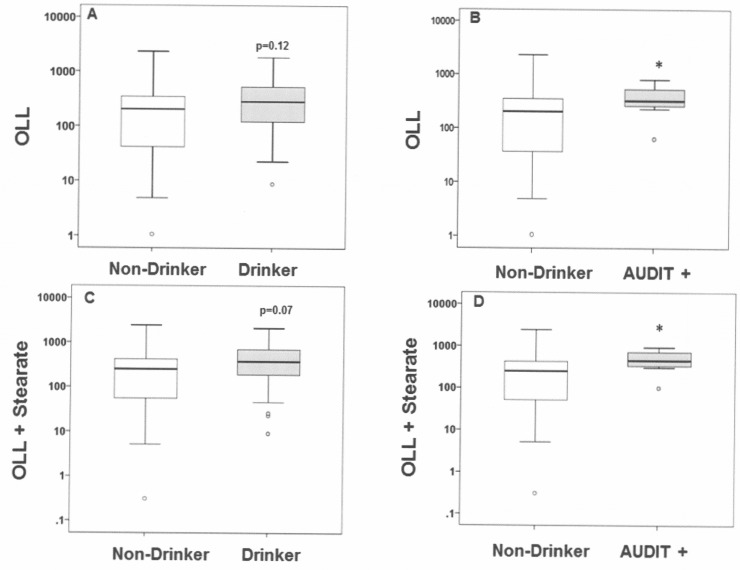
Combinations of FAEEs in placental tissue. The Y axis represents the placental FAEE combinations (μg/gm dry weight in logarithmic scale) of OLL (Panels A and B) and OLL + Stearate (Panels C and D) while the X axis denotes maternal drinking groups. The box plots depict the median line and the first and third quartiles are represented by the lower and upper box edge, respectively. The whiskers indicate the smallest and largest values measured with outliers depicted by a small circle. * denotes p ≤ 0.05 AUDIT+ versus Non-Drinker.

Accumulation of the combination of unsaturated 18 carbon FAEEs (such as ethyl oleate + linoleate + linolenate, designated OLL) as well as the saturated 18 carbon ethyl stearate has been described in multiple alcohol-exposed organs, meconium, and other samples (such as hair) in guinea pig [25,26] and mouse models [7]. Therefore, we similarly evaluated the combinations of OLL and OLL + Stearate in the placental samples to determine if they were significantly elevated with different patterns of maternal alcohol use (Fig 1).

The combinations of OLL and OLL + Stearate were elevated but did not reach significance in Drinkers versus Non-Drinkers (Fig 1. Panels A and C). However, the combinations of OLL and OLL + Stearate were each significantly elevated in the AUDIT+ placentas compared to Non-Drinkers (Fig 1. Panels B and D).

### ROC analyses of FAEEs

**Fig 2:**
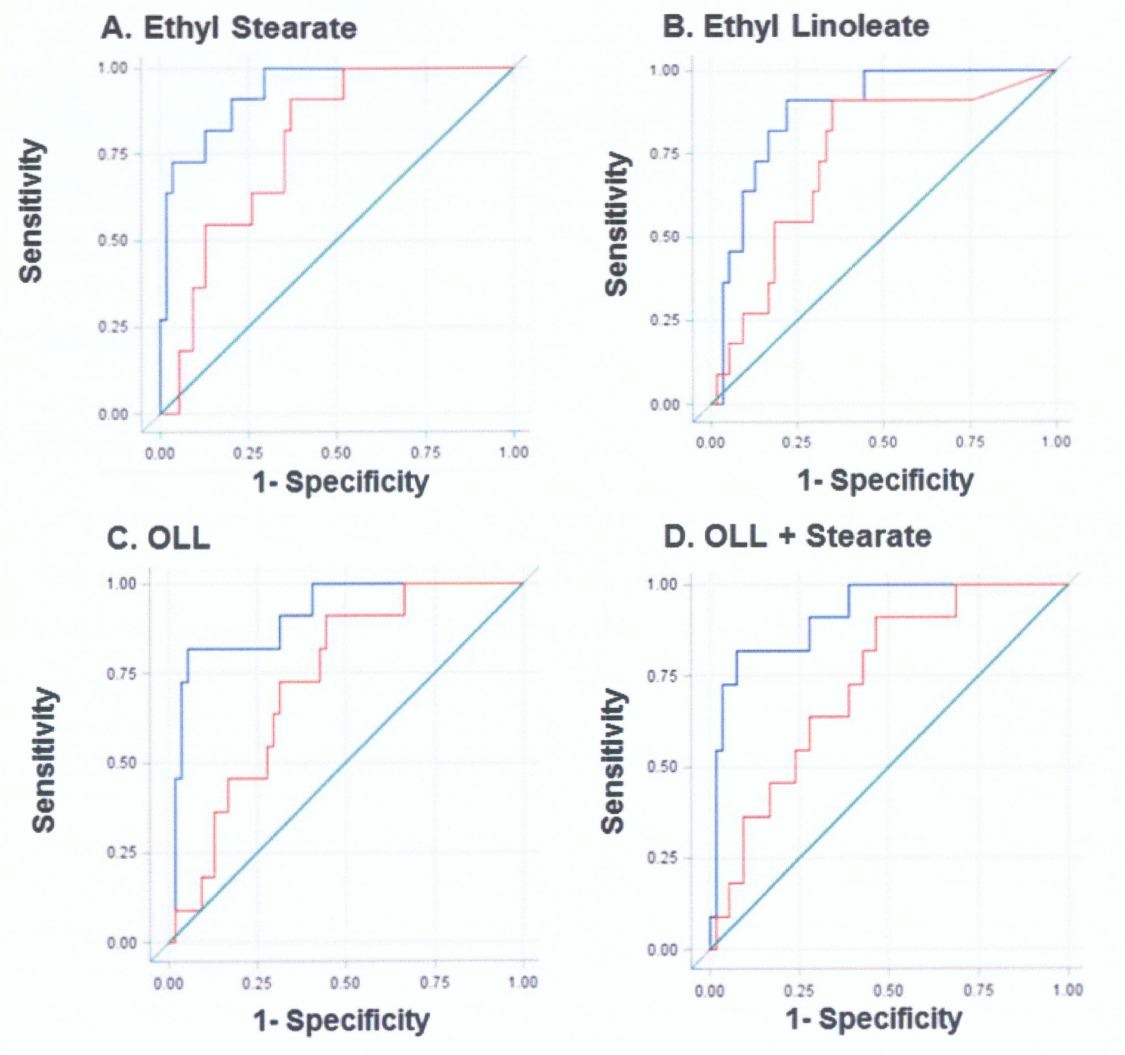
Receiver Operating Characteristic curves for AUDIT+. Unadjusted and adjusted ROC curves were generated for (A) Ethyl Stearate, (B) Ethyl Linoleate, (C) OLL and (D) OLL + Stearate. The Y axis denotes Sensitivity and the X axis denotes 1- Specificity. The red line denotes the unadjusted curve and the blue line denotes the curve adjusted for gestational age, maternal smoking and illicit drug use. A green diagonal reference line (line of no discrimination) corresponds to an area under the curve (AUC) of 0.5.

We generated ROC curves for individual and combinations of FAEEs to assess the relationship between FAEEs levels and alcohol exposure in order to evaluate if FAEEs could significantly predict AUDIT+ pregnancies. Since maternal smoking and illicit drug use were higher in those who reported drinking, and gestational age weakly correlated with FAEEs, we evaluated both unadjusted ROC curves and curves adjusted for gestational age, maternal smoking, and illicit drug use (Fig 2).

Adjusted ROC analyses demonstrated that the individual FAEEs ethyl stearate and ethyl linoleate and the combinations of OLL, OLL + Stearate generated AUC ranging from 88–93%, with sensitivities of 82% and specificities ranging from 83–94%. Positive predictive values (PPV) ranged from 44–70% while negative predictive values (NPV) were 97%. Ethyl Stearate proved the strongest placental marker, with the highest area under the curve of 93%, sensitivity of 82%, specificity of 87%, positive predictive value of 50% and negative predictive value of 97% (Table 3).

**Table 3:** Analysis of Adjusted ROC Curves for AUDIT+.

	AUC (95%CI)	p	Sensitivity (%)	Specificity (%)	PPV (%)	NPV (%)
**Ethyl Stearate**	0.933 (0.864–1.000)	0.0001	82	87	50	97
**Ethyl Linoleate**	0.877 (0.782–0.972)	0.0001	82	83	44	97
**OLL**	0.911 (0.819–1.000)	0.0001	82	94	70	97
**OLL + Stearate**	0.918 (0.832–1.000)	0.0001	82	93	64	97

AUC- area under curve; PPV- positive predictive value; NPV-negative predictive value.

Our data demonstrated that maternal admission of alcohol use during pregnancy occurred in approximately one third of premature pregnancies, with 5% reporting binge drinking during pregnancy. Our data mirrors previous reports by Lester *et al* for this birth weight category [27], as well as the National Birth Defects Prevention Study, of which Georgia was a participating state. In this study, 30% of women drank at some time during their pregnancy while ~8% binge drank. [28] We also identified that 14% of the subjects (approximately 1 in 7) were suspected to have problematic drinking based on positive AUDIT responses. However, we suspect this under-represents the true use of alcohol during pregnancy, particularly given that excessive maternal drinking can increase the risk of extreme prematurity [14,29,30,31] and the known limitations of using questionnaires to identify alcohol exposure [15,16,32]. Despite the limitations of the questionnaire, and the lack of a gold standard to identify alcohol-exposed pregnancies, we have no evidence that subjects would report alcohol use or problematic drinking if they indeed did not drink alcohol; thus misclassification of subjects would likely obscure real findings, rather than amplify exposure. Therefore, 30% alcohol exposure and 14% problematic drinking are valid estimates in these pregnancies that ended prematurely, and support that maternal drinking increases the risk of extreme preterm delivery.

Importantly, there were no clinical characteristics of the studied subjects which could differentiate those who consumed alcohol during pregnancy or had problems with alcohol use from those who did not, short of cigarette smoking during pregnancy, as has been described by others [27,33]. Specifically, there were no differences in maternal age, race distribution, maternal education, maternal income, rates of prenatal care, delivery route or gestational age in the alcohol-exposed pregnancies compared to un-exposed pregnancies. Therefore, clinically identifying which premature delivery was alcohol-exposed using such routine parameters remains inaccurate. Thus, accurately identifying the alcohol-exposed premature newborn admitted to the Newborn Intensive Care Unit continues to be clinically challenging and exposed premature newborns remain clinically undetected.

Multiple biomarkers of fetal alcohol exposure including FAEEs [5,6,7,8], ethyl glucuronide [34], and ethyl sulfate [35,36] in meconium, hair, and placenta [37] have been shown to correlate with alcohol exposure and the development of fetal alcohol spectrum disorder at term gestation [38,39]. Although FAEEs have been demonstrated in multiple organs of experimental animals exposed to alcohol, including the placenta [7], this is the first report describing FAEEs in human placental tissue in attempts to identify alcohol exposure in pregnancies ending prematurely. We demonstrated increased levels of ethyl palmitate in Black subjects compared to White subjects. This requires further investigation particularly given the described disparities in the effects of alcohol toxicity and the increased risk of fetal alcohol syndrome in Blacks. [40] Our data demonstrated that multiple placental FAEEs including ethyl stearate and ethyl linoleate, and combinations of FAEEs such as OLL and OLL + stearate were elevated and demonstrated high sensitivity in those pregnancies with self-reported maternal drinking, particularly in the AUDIT+ group. Furthermore, placental FAEEs demonstrated high negative predictive values (NPVs) indicating that if placental FAEEs fell below a particular cut off, then the premature pregnancy could be identified as highly unlikely to be alcohol-exposed. Such high NPVs have similarly been demonstrated in the initial studies of meconium FAEEs analyses used to identify the alcohol-exposed term newborn [5].

There are several limitations of this study. We were unable to eliminate variability in the timing of the placental sampling. Studies in meconium have demonstrated that a delay in collection of meconium can introduce the possibility of increased FAEE measurements due to the presence of microbes and substrates which can themselves produce FAEEs [41]. However, studies have also demonstrated that FAEEs are directly metabolized by the placenta and can diminish over time [9]. This raises the possibility that our measurements may have underestimated the FAEEs concentration in the placental samples. Our sample size was insufficient to perform a more vigorous subgroup analysis for the potential relationship between the exact amounts of alcohol ingested during the pregnancy and placental FAEEs. Furthermore, given the focus of evaluating only pregnancies ending prematurely, additional studies are necessary to determine whether our findings are applicable to alcohol-exposed term pregnancies. In studying only premature deliveries, we were unable to fully evaluate the potential effects of advancing gestational age on the accumulation of placental FAEEs with maternal drinking during pregnancy.

## Conclusions

We conclude that one third of pregnancies ending prematurely are alcohol exposed. This is the first report demonstrating elevation of placental FAEEs in alcohol exposed premature pregnancies. Placental analysis for biomarkers of alcohol exposure such as FAEEs holds great promise to accurately identify the at-risk pregnancy, including those ending prematurely. Since maternal self-report underestimates alcohol use by 4 fold compared to biomarker analyses, [32] additional matrices such as the placenta may add clinically important information regarding alcohol exposure and its potential toxicity to the newborn. We have recently demonstrated direct cellular toxicity due to FAEE exposure. [21] However, as recently noted in a review regarding FAEEs, [42] the direct toxicity of FAEEs to the placenta as well as alcohol-exposed fetal tissues requires further investigation. Until *in utero* alcohol exposure can be accurately identified in premature newborns, we will not fully understand its consequences on this vulnerable population. Continued investigations into the use of placental tissue as an additional source for accurate identification of the alcohol-exposed pregnancy are warranted.
