# Correction: Placental Fatty Acid Ethyl Esters Are Elevated with Maternal Alcohol Use in Pregnancies Complicated by Prematurity

**DOI:** 10.1371/journal.pone.0136366

**Published:** 2015-08-19

**Authors:** Theresa W. Gauthier, Sowmya S. Mohan, Teresa S. Gross, Frank L. Harris, David M. Guidot, Lou Ann S. Brown

The second affiliation for author David M. Guidot is not indicated. Dr. Guidot is also affiliated with: Atlanta VA Medical Center, Decatur, GA 30033
